# Prenatal findings of cataract and arthrogryposis: recurrence of cerebro-oculo-facio-skeletal syndrome and review of differential diagnosis

**DOI:** 10.1186/s12920-021-00939-6

**Published:** 2021-03-25

**Authors:** Fabio Sirchia, Ilaria Fantasia, Agnese Feresin, Elisa Giorgio, Flavio Faletra, Denise Mordeglia, Moira Barbieri, Valentina Guida, Alessandro De Luca, Tamara Stampalija

**Affiliations:** 1 Department of Molecular Medicine, University of Pavia, Via Forlanini 14, 27100 Pavia, Italy; 2 Unit of Fetal Medicine e Prenatal Diagnosis, Institute for Maternal and Child Health IRCCS “Burlo Garofolo”, Trieste, Italy; 3 Department of Medicine, Surgery and Health Sciences, University of Trieste, Trieste, Italy; 4 Department of Medical Sciences, University of Torino, Turin, Italy; 5 Department of Medical Genetics, Institute for Maternal and Child Health IRCCS “Burlo Garofolo”, Trieste, Italy; 6 Division of Medical Genetics, Fondazione IRCCS-Casa Sollievo della Sofferenza, 71013 San Giovanni Rotondo, Foggia, Italy

**Keywords:** COFS3, *ERCC5* gene, Arthrogryposis, Fetal growth restriction, Case report

## Abstract

**Background:**

Cerebro-oculo-facio-skeletal syndrome (COFS) is a severe and progressive neurologic condition characterized by prenatal onset of arthrogryposis, cataract, microcephaly and growth failure. The aim of this study was to present a case of recurrence of the COFS syndrome and to propose a differential diagnosis flow-chart in case of prenatal findings of arthrogryposis and cataract.

**Case presentation:**

We report a case of recurrence of COFS3 syndrome within the same family, with similar diagnostic features. In the first case the COFS syndrome remained undiagnosed, while in the second case, due to prenatal findings of arthrogryposis and cataract, genetic investigation focusing on responsible genes of COFS (*ERCC5*, *ERCC6* and *FKTN* genes) was carried out. The fetus was found to be compound heterozygous for two different *ERCC5* mutations, confirming the clinical suspect of COFS syndrome. A review of the literature on possible causative genes of prenatal cataract and arthrogryposis was performed and we present a flow-chart to guide differential diagnosis and possible genetic testing in case of these findings.

**Conclusion:**

COFS syndrome is a rare autosomic recessive condition. However, it can be suspected and diagnosed prenatally. The flow-chart illustrates a pathway to guide differential diagnosis according to the prenatal findings. Main syndromes, key testing and specific genes are included. Targeted molecular testing should be offered to the couple in order to reach a diagnosis and assess the recurrence risk for future pregnancies.

## Background

Cerebro-oculo-facio-skeletal (COFS) syndrome is the extreme expression of Cockayne syndrome with prenatal manifestations. Typical clinical picture of COFS includes congenital microcephaly, cataract, microphthalmia, arthrogryposis, severe prenatal and postnatal growth failure, axial hypotonia, sensorineural deafness and facial dysmorphisms with prominent nasal root and/or overhanging upper lip. COFS can cause intrauterine fetal death or, alternatively, severe psychomotor retardation is constant and death usually occurs in the first months/years of life [1, 2].

COFS is a rare genetic disorder, although the real incidence is unknown, with an autosomal recessive inheritance pattern and it is caused by mutations in different genes encoding components of the machinery of nucleotide excision repair (NER), a DNA repair mechanism [1, 3].

At the moment, four different genes are known to cause COFS syndrome: *ERCC6* (COFS1, MIM #214150), *ERCC2* (COFS2, MIM #610756), *ERCC5* (COFS3, MIM #616570), and *ERCC1* (COFS4, MIM #610758) [3, 4]. Biallelic mutations in *ERCC5* gene are responsible for the type 3 of the syndrome, also known as COFS3. The *ERCC5* gene codifies for a structure-specific endonuclease required for making the 3-prime incision during DNA nucleotide excision repair and, therefore, contributes to eliminate a broad spectrum of structural DNA lesions [5]. Biallelic mutations in this gene have been associated with different phenotypes from xeroderma pigmentosum (XPG), where manifestations are largely restricted to the skin, to trichothiodystrophy, Cockayne syndrome and the more severe, early-onset COFS3 [6].

## Case presentation

We report a case of recurrence of COFS3 syndrome within the same family with similar diagnostic features. Parents were healthy, young (< 30 years) and coming from different countries. Consanguinity was therefore excluded. No cases of congenital malformations or recurrent diseases were present in their families.

**Fig. 1:**
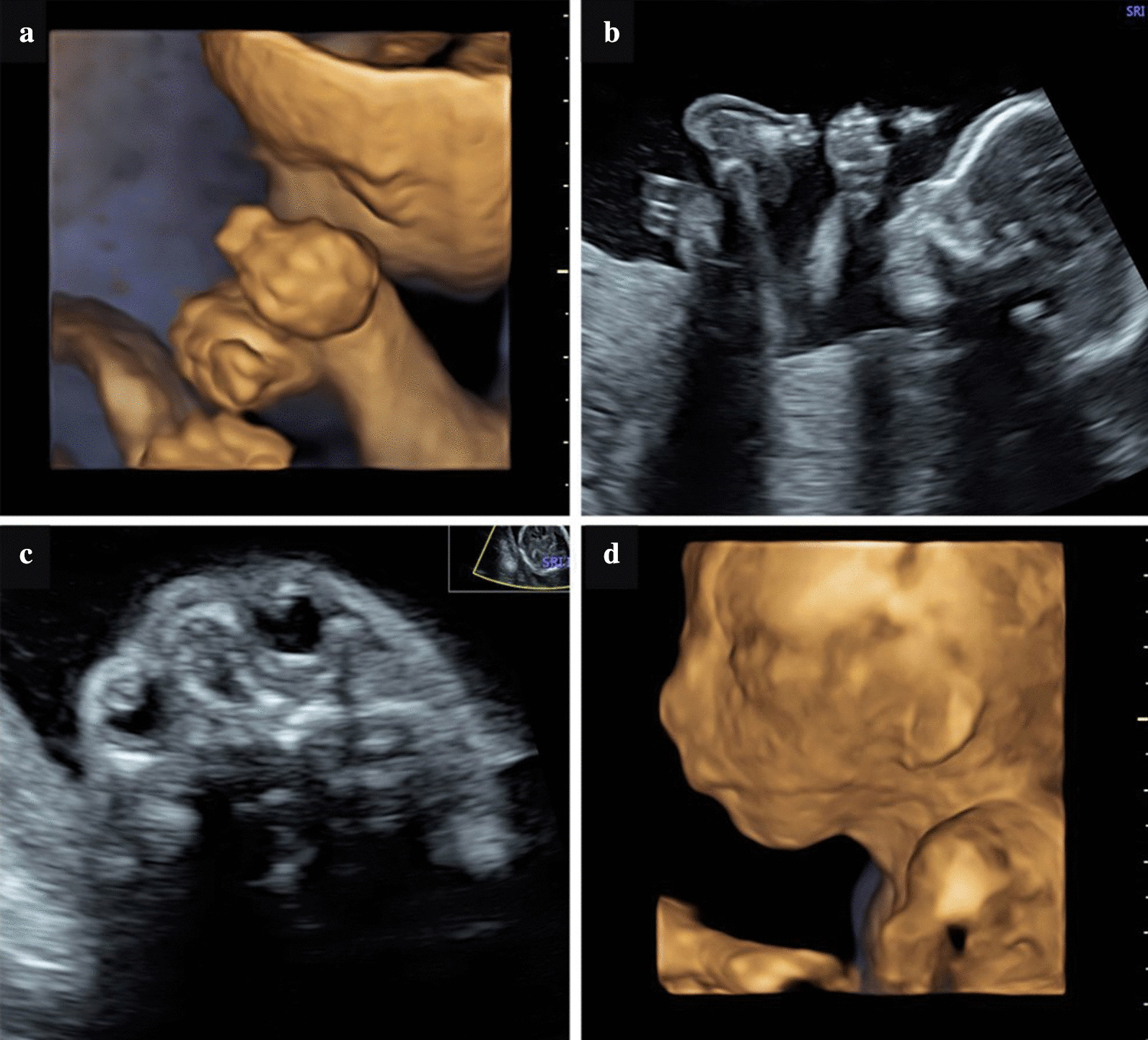
The figure represents the prenatal findings at 2D and 3D ultrasound of Case 2: **a** clenched hand with abducted fingers; **b** rockerbottom foot with plantar edema; **c** bilateral microphthalmia with cataracts; and **d** micrognathia and low-set ears

- *Case 1* The first pregnancy, followed-up in another hospital, was conceived spontaneously and was reported to be uneventful for the first 5 months until the diagnosis of fetal growth restriction (FGR) due to fetal biometry below the 3rd centile, and ventriculomegaly confirmed by fetal magnetic resonance imaging (MRI). A cesarean section was performed at 31 weeks’ gestation due to severe FGR. At birth, a female baby weighted 930 g (< 3rd centile), was 33 cm (<< 3rd centile) in length and with a head circumference of 27.5 cm (3rd–10th centile). The APGAR score was 7/8/8 at the 1′, 5′ and 10′ min of life, respectively. The physical examination showed some peculiar facial dysmorphisms, including microphthalmia, high nasal root and micrognathia. She presented with congenital generalized arthrogryposis with multiples contractures and hypertonia. The baby needed oxygen supplementation for respiratory distress and was then admitted to the Neonatal Intensive Care Unit. The neonatal MRI confirmed the ventriculomegaly and detected a hypoplastic cerebellar vermis. In addition, there was a cataract in the right eye, while fundus oculi were bilaterally normal. Abdominal ultrasound showed hyperechogenic kidneys. The electrocardiogram and the echocardiogram were normal with a physiologically patent foramen ovale. She presented with feeding difficulties, requiring positioning of a feeding-tube. Severe failure to thrive was due to worsening of general condition. She died because of superimposed kidney failure and pulmonary haemorrhage at 2 months of life. The family refused autopsy. Postnatal conventional karyotype and array comparative genomic hybridization (array-CGH) were performed on the proband and resulted both negative. No specific diagnosis was made, and the case was concluded as a likely rare syndrome of unknown origin. A second pregnancy of the couple resulted in a healthy live born male child.
- *Case 2* A third pregnancy was spontaneously conceived some years later. The first trimester scan was normal, with a nuchal translucency of 1.2 mm and a low risk for major aneuploidies at combined test. At 16 weeks, fetal biometry and anatomy were normal. The couple was referred to our hospital at 21 weeks because of the recurrence of FGR. At ultrasound examination FGR was confirmed and multiple morphological abnormalities, similar to those of the first deceased baby, were reported: the hands were clenched during the whole time of the examination with abducted fingers (Fig. 1a); there was a right rocker-bottom foot with plantar edema (Fig. 1b); both superior and inferior limbs were constantly extended with no evidence of flexion-extension at joints; the lenses were bilaterally hyperechoic, suggestive for cataract, and the orbital diameter was bilaterally below the 5th centile (Fig. 1c); slight retrognathia and low implantation of the ears were also reported (Fig. 1d). A multidisciplinary counselling was performed during which the parents were informed regarding the poor prognosis; the likelihood of a hereditary condition and the possibility of a COFS syndrome were hypothesized. The amniocentesis was performed. As first-tier genetic test, we performed a single nucleotide polymorphism (SNP’s) array analysis (ILLUMINA Express Exome arrays) and metabolic investigations, including the measurement of 7-dehydrocholesterol, in order to detect any chromosomal molecular abnormality and to exclude the Smith–Lemli–Opitz Syndrome (SLOS).

**Fig. 2:**
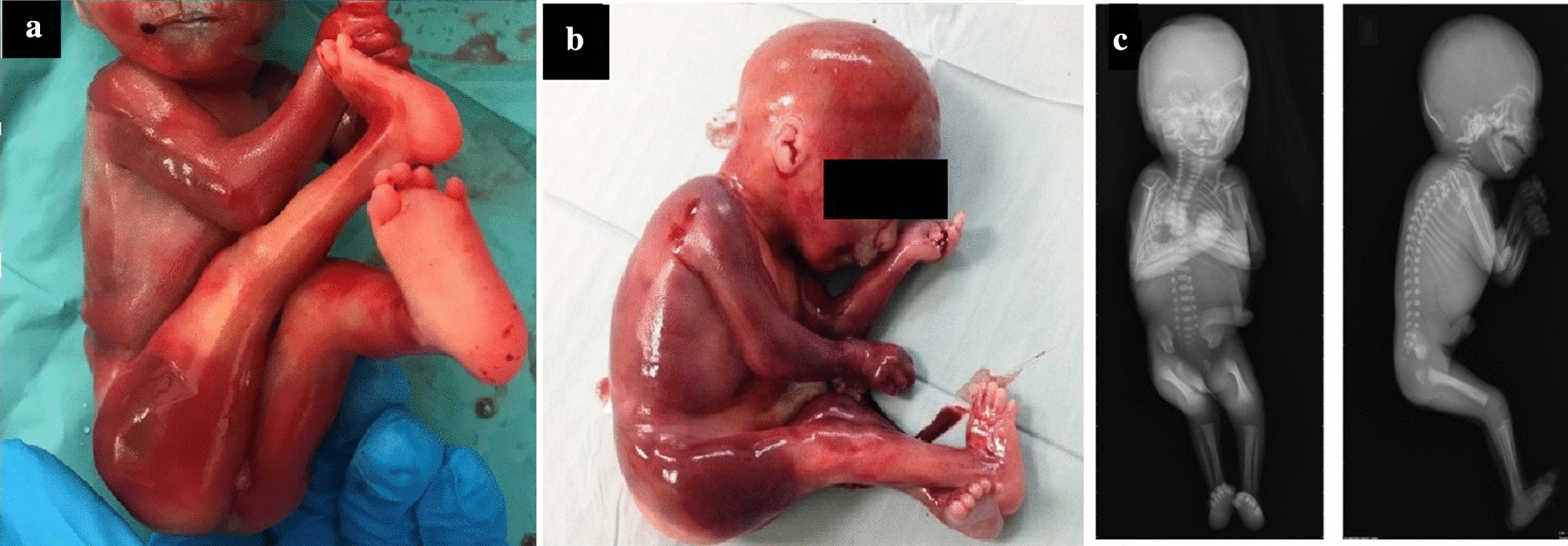
The figure shows the fetal external examination: **a** upper and lower limb abduction; **b** thumbs adduction; and **c** radiological post-mortem findings

The couple elected for a termination of pregnancy. The fetus underwent an external macroscopic examination followed by an autopsy: prenatal findings were confirmed, and absent palmar and plantar creases were detected (Fig. 2a, b). Post-mortem radiological investigations (skeletal X-ray, total body computed tomography (CT) and MRI) were performed: X-ray confirmed the contracture of the upper and lower limbs (Fig. 2c), and no additional findings were encountered.

The SNP’s array analysis and metabolic investigations resulted negative. Due to the prenatal findings, a COFS syndrome was suspected and further genetic investigation focusing on responsible genes were carried out: *ERCC5, ERCC6* and *FKTN* genes (candidate genes included in the ILLUMINA Trusightone Clinical Exome Sequencing panel). The analysis of *ERCC5* gene (NM_000123.3) revealed the presence of two mutations in compound heterozygosity (confirmed by the segregation in the parents): c.1096C>T (p.Arg366*) on the paternal allele and c.2269C>T (p.Gln757*) on the maternal allele. Both mutations result in a premature stop codon, and in a truncated and likely nonfunctional protein product, confirming the clinical suspicion of COFS3.

## Discussion and conclusions

The present literature on the prenatal diagnosis of COFS syndrome is sparse and mainly related to case-series. The first published case-report was reported by Paladini et al. [7] in a fetus with bilateral severe fetal microphthalmia, micrognathia and moderate contractures of the upper and lower limbs in a patient with a previous obstetric history suggestive for COFS syndrome recurrence. The diagnosis was made by clinical presentation only and no genetic test was performed. Only 8 cases from three different families were described with a prenatal presentation of COFS3 [2, 8]. All pregnancies were terminated because of the abnormal ultrasound findings. In these cases, the findings included joint contractures, microcephaly, micrognathia, cerebral and ocular abnormalities. Particularly, ocular findings were reported only in three cases reported by Le Van Quyen et al. [8] who described the presence of cataract and at least one other ocular finding. However, the importance of findings such as microphthalmia or cataract is corroborated by postnatal series where these are reported to be present in the large majority of children affected by COFS syndrome [2, 3].

To our knowledge, no other cases with prenatal suspicion and genetic diagnosis of the other types of COFS (COFS1, COFS2 or COFS4) are reported in literature. Most likely this might be a consequence of missed diagnosis, since the vast majority of the cases described presented with cataract and/or microphthalmia and arthrogryposis at birth [1, 5].

Arthrogryposis multiplex congenita (AMC) is a rare condition characterized by two or more major contractures in different body areas. The etiology is highly heterogeneous and prenatal diagnosis is reached in around 25% during an obstetric routine care [9, 10]. Apart from a recent Consult Series [11], there is a lack of standardized protocols that may help the clinician in the approach of the prenatal diagnosis of AMC. Finges et al*.* propose an interesting flow-chart that mainly divides AMC associated to additional anomalies or AMC limited to limbs: in the associated anomalies group, they include brain anomalies, microcephaly, ventriculomegaly with no mention of ocular abnormalities. As stated by the authors, the value of genetic testing is questionable if the approach to AMC is not systematic since more than 400 syndromes are associated to AMC. Microarray analysis is recommended as the first-tier genetic test, while targeted molecular testing is indicated when a family history is suggestive for a single-gene defect or when multiple abnormalities are present [9, 10].

In case of a syndromic fetus, it is of extreme importance to identify distinctive features that may guide the clinician towards the specific diagnostic test(s). We think that microphthalmia/cataract may play a role in this direction in case of COFS syndrome, but differential diagnosis with other conditions is however important.

Microphthalmia is present in multiple genetic syndromes, and cataract may also be associated to infectious diseases. However, both findings are rarely associated to arthrogryposis. Together with other signs, such as 2–3 toe syndactyly, postaxial polydactyly or genital anomalies, microphthalmia/cataract should prompt suggest SLOS. Moreover, cataract is an uncommon finding at the second trimester ultrasound, and it is present at birth only in the 20% of SLOS patients [11, 12].

Micrognathia is frequently associated to AMC, but it is not of help in the differential diagnosis since it is a consequence of decreased fetal activity/movements often associated with FGR, polyhydramnios, pulmonary hypoplasia and short umbilical cord. These are the key signs of the Fetal Akinesia Deformation Sequence (FADS) also known as Pena-Shokeir syndrome. Many underlying causes of FADS have been recognized including genetic, maternal and environmental factors, resulting in a complex diagnostic framework [13, 14].

Microcephaly has been reported as the most represented feature in COFS syndrome, however, it is not specific and could be misinterpreted as a general FGR finding [8]. The association of cataract and arthrogryposis is also present in primary microcephaly-10 (MCPH10), an autosomal recessive disorder caused by biallelic mutations in *ZNF335* and characterized by extremely small head size (from − 3 to − 9 SD) with brain malformations [15]. In this case, the presence of microcephaly should guide the differential diagnosis with COFS syndrome.

Cerebral abnormalities, like abnormal sulci or posterior fossa defects, have also been reported in association with COFS syndrome [2]. However, reduced fetal movements and the fixed fetal position may prevent the study of these anatomical regions by ultrasound and misguide in the diagnostic process, while fetal MRI could increase the diagnostic accuracy of these defects [16, 17].

**Fig. 3:**
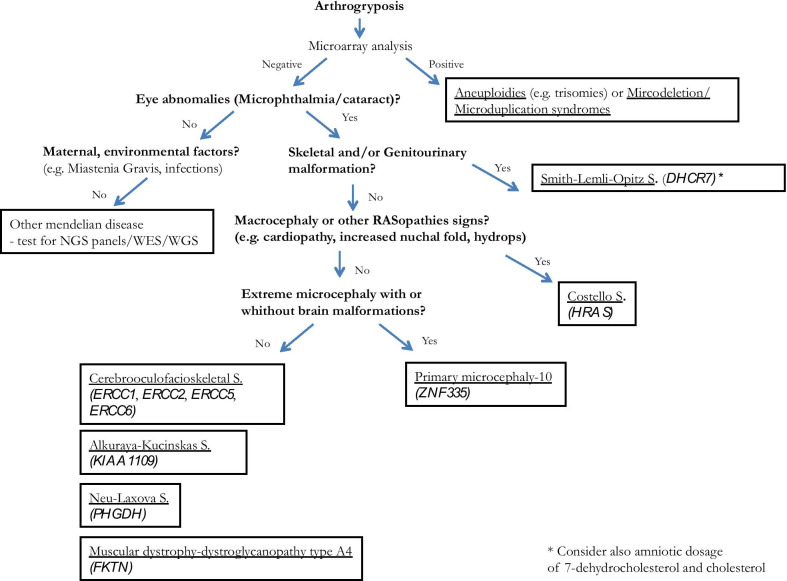
The flow-chart illustrates a possible pathway to guide differential diagnosis according to possible prenatal findings starting from arthrogryposis. Main syndromes, key testing and specific genes are included

The observed combination of cataract, arthrogryposis and microcephaly is also reminiscent of the Alkuraya-Kucinskas Syndrome (ALKKUCS) and Neu–Laxova syndrome, autosomal recessive disorders characterized by mutations in *KIAA1109* and *PHGDH* genes, respectively. Both syndromes present a strong overlap with COFS syndrome [18]. Finally, the most severe forms of congenital muscular dystrophy-dystroglycanopathy with brain and eye anomalies type A4 (MDDGA4), a rare muscular dystrophy caused by biallelic mutations in *FKTN*, can present with prenatal cataract, microphthalmia and arthrogryposis [19]. Since in these last three cases the differential diagnosis with COFS is difficult due to the possible absence of other ultrasound signs, we suggest considering the analysis of *KIAA1109*,* PHGDH* and *FKTN* together with COFS specific genes when arthrogryposis is associated with ocular signs such as cataract and/or microphthalmia. In Fig. 3 we propose a flow-chart based on the discussed review to guide differential diagnosis when cataract and arthrogryposis are encountered.

In conclusion COFS is a rare, autosomic recessive condition, which can show detectable prenatal signs. The association of microphthalmia and/or cataract with arthrogryposis seems to be peculiar to COFS syndrome and it is a rare association described in few other conditions. In the genomic era, molecular diagnosis is possible, and it is crucial to estimate the family recurrence risk. We suggest including a detailed assessment of the fetal eyes and lenses when the presence of limbs abnormalities raises the suspicion of AMC. In addition to array-analysis, targeted molecular testing for the 46 NER genes (including *ERCC1*, *ERCC2*, *ERCC5* and *ERCC6*), *KIAA1109, PHGDH* and *FKTN* genes should be offered to the couple in order to reach a diagnosis and assess the recurrence risk for future pregnancy.

## Data Availability

The raw data of whole-exome sequencing of the patient in this study are not publicly available in order to protect participant confidentiality but are available from the corresponding author on reasonable request. Reference sequences for *ERCC5* are available in the following repository (https://www.ncbi.nlm.nih.gov/nuccore/166795290). Databases used in this study were Human Gene Mutation Database (HGMD, http://www.hgmd.cf.ac.uk), ClinVar database (https://www.ncbi.nlm.nih.gov/clinvar), dbSNP (https://www.ncbi.nlm.nih.gov/snp/), gnomAD Browser (https://gnomad.broadinstitute.org/), SIFT (http://provean.jcvi.org/index.php), PolyPhen-2 (http://genetics.bwh.harvard.edu/pph2/), and Mutation Taster (http://www.mutationtaster.org/).

## Abbreviations

COFS: Cerebro-oculo-facio-skeletal syndrome
NER: Nucleotide excision repair
XPG: Xeroderma pigmentosum
FGR: Fetal growth restriction
MRI: Magnetic resonance imaging
CGH: Comparative genomic hybridization
SNP: Single nucleotide polymorphism
SLOS: Smith–Lemli–Opitz syndrome
CT: Computed tomography
AMC: Arthrogryposis multiplex congenita
FADS: Fetal Akinesia Deformation Sequence
MCPH10: Primary microcephaly-10
ALKKUCS: Alkuraya-Kucinskas Syndrome
MDDGA4: Muscular dystrophy-dystroglycanopathy with brain and eye anomalies type A4
